# Biomechanical Effects of Adding an Ankle Soft Actuation in a Unilateral Exoskeleton

**DOI:** 10.3390/bios12100873

**Published:** 2022-10-14

**Authors:** Sophia Otálora, Felipe Ballen-Moreno, Luis Arciniegas-Mayag, Carlos A. Cifuentes, Marcela Múnera

**Affiliations:** 1 Graduate Program of Electrical Engineering, Federal University of Espirito Santo, Vitoria 29075-910, Brazil; 2 Robotics & Multibody Mechanics (R&MM) Research Group, Department of Mechanical Engineering, Vrije Universiteit Brussel, 1050 Brussels, Belgium; 3 Flanders Make, 1050 Brussels, Belgium; 4 Bristol Robotics Laboratory, University of the West of England, Bristol BS16 1QY, UK; 5 School of Engineering, Science and Technology, Universidad del Rosario, Bogota 111711, Colombia; 6 Department of Biomedical Engineering, Colombian School of Engineering Julio Garavito, Bogota 111166, Colombia

**Keywords:** EMG analysis, exoskeleton, orthosis, rehabilitation, stroke

## Abstract

Stroke disease leads to a partial or complete disability affecting muscle strength and functional mobility. Early rehabilitation sessions might induce neuroplasticity and restore the affected function or structure of the patients. Robotic rehabilitation minimizes the burden on therapists by providing repetitive and regularly monitored therapies. Commercial exoskeletons have been found to assist hip and knee motion. For instance, unilateral exoskeletons have the potential to become an effective training system for patients with hemiparesis. However, these robotic devices leave the ankle joint unassisted, essential in gait for body propulsion and weight-bearing. This article evaluates the effects of the robotic ankle orthosis T-FLEX during cooperative assistance with the AGoRA unilateral lower-limb exoskeleton (hip and knee actuation). This study involves nine subjects, measuring muscle activity and gait parameters such as stance and swing times. The results showed a reduction in muscle activity in the Biceps Femoris of 50%, Lateral Gastrocnemius of 59% and Tibialis Anterior of 35% when adding T-FLEX to the AGoRA unilateral lower-limb exoskeleton. No differences were found in gait parameters. Nevertheless, stability is preserved when comparing the two legs. Future works should focus on evaluating the devices in ground tests in healthy subjects and pathological patients.

## 1. Introduction

Ischemic stroke is caused by an abrupt obstruction of blood flow to a part of the central nervous system [1]. Stroke is one of the leading causes of mortality and disability worldwide [2]. In Colombia, stroke prevalence rate was 6.5 per 1000 inhabitants, with a considerable proportion of cases going undiagnosed or untreated, resulting in partial or complete disabilities [3]. Stroke patients mainly present disabilities as changes in emotional function, muscle strength and mobility. Falling risks are also observed during functional exercises, which requires continuous medical attention from rehabilitation professionals [4].

Commonly, patients with stroke suffer difficulties performing activities of daily living due to their lack of mobility [5]. This occurs because they present in some cases hemiparesis where one-sided weakness is observed [6]. This type of pathology can be treated through physical and robotic therapy where it has been found that the recovery mechanism is more effective in the first three months [7] with the restoration of neurological functions [8] and the development of activities of daily living [9].

These rehabilitation approaches allow to recover the motor control and the person’s autonomy can be restored [10]. In the case of physical rehabilitation, therapists require more effort to assist the patient [11], whereas robotic rehabilitation minimizes the physical burdens on the therapist through repetitive and high-intensity therapies where the patient’s progress is monitored [12].

Currently, in rehabilitation robotic technologies are wearable robotic exoskeletons and robotic orthoses that can control the movements of different joints in the training process providing power and assistance [13]. Exoskeletons that have received FDA approval are the ReWalk [14], EksoNR [15] and HAL for people with different neurological diseases. Likewise, there are unilateral exoskeletons for hip, knee and ankle joints such as KAD [16] which have been validated for their potential to become an effective training system for patients with kinematic and physiological improvements. In addition, there are multiple ankle rehabilitation robots that allow long-term rehabilitation such as parallel ankle rehabilitation robots (PARR) due to the importance of the ankle in balance, support and propulsion [17]. Finally, there are also 3-joint modular exoskeletons for design [18], control and evaluation [19], and energy efficiency [20], which allows the activation of different joint configurations according to the application requirement.

However, commercial and unilateral exoskeletons have a limitation concerning the ankle performance because some of them lack it or have it as a rigid mechanism which causes the joint to have no rotational movement and restricts the range of motion when walking [21]. Further, modular exoskeleton studies do not show a comparison between their different modules or the biomechanical effect of adding ankle actuation in the same exoskeleton. The ankle joint provides shock absorption and acts as a rigid segment that generates body propulsion in the toe-off phase during gait [22]. For this reason, the performance of this joint is necessary for walking with an exoskeleton because the ankle joint supports the weight of the entire exoskeleton and helps with the propulsive force when walking [23].

The AGoRA unilateral lower-limb exoskeleton is a structure with rigid actuators located at the center of rotation of the hip and knee joints allowing flexion and extension movement of these joints and passive hip movement in adduction/abduction [24]. It has two control strategies, namely, transparency and assistance mode. In the transparency mode, the user’s force/torque is considered and converted to angular velocities. In assistance, torques in the range of 20 Nm to 30 Nm are generated at the hip and knee joints where the degree of assistance can be varied [25]. In addition, the exoskeleton has been evaluated for human–robot interaction (HRI) using a three-dimensional relative motion method. Six healthy subjects were evaluated in 6-m walking tests where the relative motion analysis showed transmission loss that can be compensated by the control strategy of the device and the design of the physical interfaces. Therefore, through the kinematic and HRI analysis of the study, the performance of the device can be improved [26].

On the other hand, the robotic orthosis, T-FLEX, is a powered ankle-foot orthosis to assist and rehabilitate ankle dysfunctions. It has two actuation systems in the frontal and posterior part of the leg. Attached to these mechanisms are tendons composed of a flexible and an elastic mechanism that performs the dorsi-plantarflexion movements of the ankle [27]. Previously, the characterization of the T-FLEX tendons was performed to evaluate the configuration with the best performance. The results show that the tendons acting without the flexible filament have greater device performance [28]. T-FLEX has the potential to complement the AGoRA unilateral lower-limb exoskeleton according to its results in stationary therapy where a long-term study has found the recovery of motor control in a stroke patient [29]. In gait activity, comparing the use and non-use of the device, an improvement in the kinematic parameters of the ankle and changes of up to 70% in the range of motion of 10 subjects was found [30].

Considering this, the main contribution of this work is the biomechanical evaluation of adding ankle actuation to a hip and knee exoskeleton by comparing 3 modes: (1) the AGoRA exoskeleton (hip and knee), (2) the integration of the T-FLEX orthosis (ankle), and (3) a baseline of the person without wearing the devices. Therefore, the analysis of these comparisons through physiological and kinematic sensory analysis can become essential in the design and development of future lower-limb exoskeletons with ankle actuation for gait assistance for people with mobility impairments. This can be observed in changes generated in muscle activity of four gait-relevant muscles and in gait parameters such as swing and stance times. The assessment was conducted in nine healthy subjects walking on a treadmill for 6 min for the three modes. Moreover, the study analyzes stability and qualitative results such as a questionnaire to observe the perception received by the users wearing the devices.

## 2. Materials and Methods

This section describes the design and control of the AGoRA unilateral lower-limb exoskeleton and the T-FLEX robotic orthosis acting together. Likewise, this section explains in detail the experimental protocol with the different stages used to evaluate the devices and the perception of the users.

### 2.1. Robotic Devices

**Figure 1:**
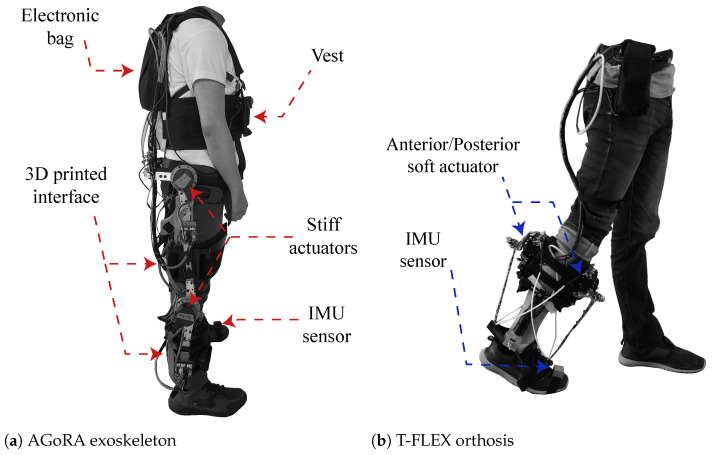
Description of the robotic devices and their main components. (**a**) Graphical illustration of the AGoRA unilateral lower-limb exoskeleton, and (**b**) T-FLEX ankle robotic orthosis.

The AGoRA unilateral lower-limb exoskeleton (see Figure 1a) is a rehabilitation device that is designed for patients with neurological diseases of the lower limb. The exoskeleton is designed with a rigid structure that comprises a duraluminium mechanical structure and a stiff actuation system located in the center of rotation of the user’s hip and knee joints [25]. The actuation system has two brushless DC electric motors (EC-60 flat 408057, Maxon Motor AG, Sachseln, Switzerland). The flexible parts that attach the device to the person are made of foam (Polyurethane 70/30, Colombia). In addition, a layer of ECOFLEX 50 silicone (Smooth-on, Macungie, PA 18062, USA) is placed on each attachment to prevent the device from slipping off the user and generating friction between the garment/skin and the device. It has a vest designed to distribute the forces of the weight of the device (20 kg) on the shoulders and the abdominal part of the user. Lastly, the gait phase detection uses an IMU sensor (BNO055, BOSCH, Gerlingen, Germany).

**Figure 2:**
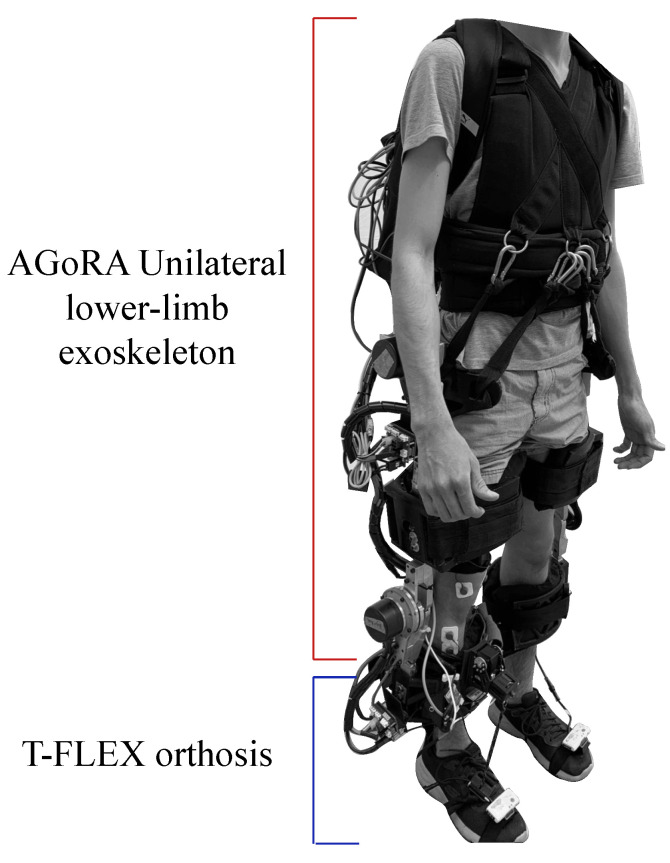
Description of the integration of the AGORA and T-FLEX exoskeletons and their main components.

The T-FLEX robotic orthosis is added to the exoskeleton (see Figure 1b), which has a flexible mechanism employing composite tendons that are coupled to the two servomotors of the device (Dynamixel MX106T, Robotis, CA 92630, USA). The tendons contain a rigid fishing rod material (eight filaments, Sufix 832, USA) and an elastic filament (Filaflex, 2.85 mm, Recreus, Spain) that produces the effect of variable stiffness to perform the dorsi-plantarflexion movement [28]. For more details concerning the structure of the devices please refer to [25] for the AGoRA Lower-limb Exoskeleton and to [28] for the orthosis T-FLEX. The coupling of the two devices is shown in Figure 2.

### 2.2. Control Strategy

The exoskeleton comprises a multilevel control design that considers the change of some parameters to assist the user’s gait cycle. This multilevel control is composed of a low-level controller that is equal to the standard controller as a PI controller. In this case, a PI controller is implemented to apply a current controller [25]. A mid-level controller is defined as an impedance controller to ease and follow to take into accordance with the Human–Robot Interaction (HRI) definition [31]. This controller allows the motion compensation of each joint using estimated torques provided by the stiff actuation system to the user [25]. Finally, the high-level controller is divided into two modules: first, the gait phase detection module that uses an IMU sensor on the tip foot to estimate four gait phases in real-time, applying the Hidden Markov Model; second, an angular position selector is implemented to change the desired position in the impedance controller according to the detected gait phase [32].

The robotic device T-FLEX is implemented to assist the movements in the ankle joint. The main goal is to apply estimated torques that complement the dorsi-plantar flexion ankle movements. T-FLEX was developed with a different concept than the AGoRA unilateral lower-limb exoskeleton, in this case, T-FLEX, lacks a rigid structure [30]. Additionally, a series Elastic Actuator (SEA) generates the estimated torques during the gait cycle [28]. This control architecture was established as a multilevel structure, where a low-level controller comprises a PID controller. This controller uses the motor position that converts into velocity and acceleration. An encoder that monitors the motor’s actual position is used to close the loop in this low-level controller. The high-level controller is composed of a gait phase detection implemented by the AGoRA unilateral lower-limb exoskeleton in this high-level controller [24]. Moreover, T-FLEX implements a states selector based on a real-time gait phase detector [28] to assist the dorsi-plantarflexion during walking. The mechatronic integration of the two devices and the control variables can be found in [33].

### 2.3. Subjects

Nine healthy male adults participated in the study. Table 1 summarizes the characteristics and information of the subjects who accomplished this study. The volunteers fulfilled the inclusion criteria: healthy adults, height between 170 and 185 cm, weight less than 110 kg, femur length between 42 and 48 cm, a hip–knee distance between 32 and 37 cm, and a knee–ankle distance between 28 and 31 cm. These are the anthropometric measurements required for the AGoRA unilateral lower-limb exoskeleton to adjust to the user’s dimensions. The exclusion criteria include: exercise intolerance, presence of wounds or ulcers, having a cognitive disability, and suffering from a pathology that prevents using an assistive gait device.

### 2.4. Experimental Setup

This protocol included three modes (i.e., without exoskeleton (WOE), with AGoRA unilateral lower-limb exoskeleton (WE) and with the AGoRA unilateral lower-limb exoskeleton and the T-FLEX orthosis (WE&T)) to analyze the effect of the T-FLEX orthosis on the AGoRA unilateral lower-limb exoskeleton. According to SENIAM guidelines, participants were instrumented with surface electrodes and an EMG acquisition module (Shimmer3 EMG Unit, Shimmer, New Bedford, MA 02139, USA).

**Figure 3:**
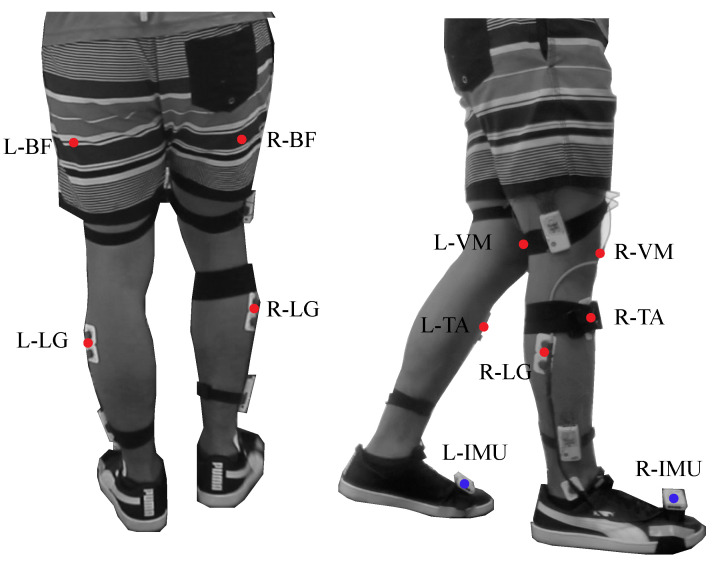
Graphical illustration of the participant’s instrumentation. The red points represent the muscles used during the device evaluation. The setup includes the following muscles: Biceps Femoris in the Right and Left leg (L−BF, R−BF), Vastus Medialis in the Right and Left leg (L−VM, R−VM), Lateral Gastrocnemius in the Right and Left leg (L−LG, R−LG) and Tibialis Anterior in the Right and Left leg (L−TA, R−TA). The blue points represent the inertial sensors in both feet.

To monitor muscle activity, four muscles relevant to the gait cycle [34] were used for the tests: Biceps Femoris (BF), Vastus Medialis (VM), Lateral Gastrocnemius (LG) and Tibialis Anterior (TA). Being BF the knee flexor muscle, VM the knee extensor muscle, LG the ankle plantar flexor and TA the ankle dorsiflexor [35,36]. Besides this, two Shimmer3 IMU (Shimmer3 IMU Unit, MA 02139, Shimmer) were located on both feet to divide the signals in gait cycles. Figure 3 shows the location and instrumentation over a participant in this study.

### 2.5. Data Analysis

**Figure 4:**
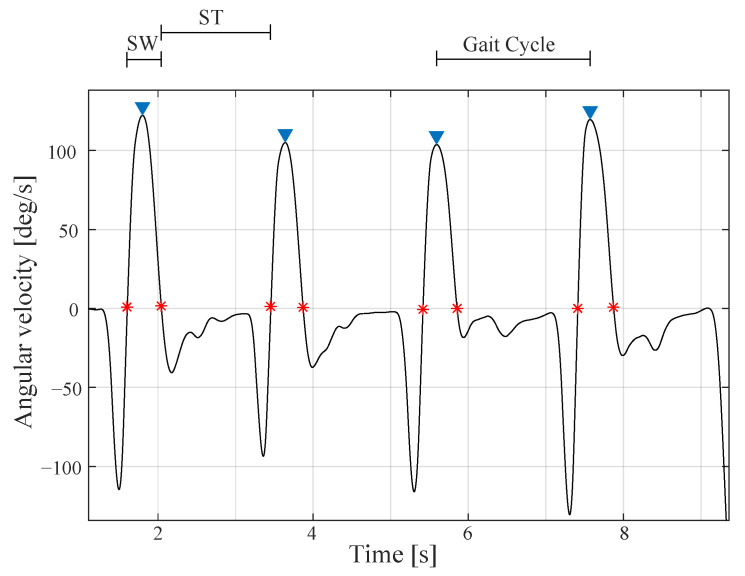
Angular velocity acquired by the toe inertial sensor. This signal is used to segment the signals into gait cycles and find swing (SW) and stance (ST) phases times. The asterisks in red indicate the initiation or the termination of the swing and stance phases.

The processing performed in the study was accomplished using MATLAB software (MathWorks, 2020a, Natick, MA, USA). Inertial sensors data was acquired with a sampling frequency of 128 Hz and was segmented and normalised to 0–100% of the gait cycle. Each gait cycle is obtained by consecutive peak heel strikes in the angular velocity of each foot as shown in Figure 4. A moving average filter was applied with a 30 ms window to identify gait events such as swing phase and stance phase. These times phases are found according to the indicators in Figure 4.

Electromyographic signals (EMG) were acquired with a sampling frequency of 1024 Hz. To extract the average linear envelope during the gait cycle, raw EMG signals were filtered with a band-pass filter to remove noise and artefact contamination [37]. Signals were filtered with a Butterworth filter with a cut-off frequency of 15 Hz to remove baseline drift usually associated with movement and removing DC offset [38,39]. Then, the signal was rectified, and the root-mean-square (RMS) value was applied with a moving average filter of 200 ms [38]. The EMG signal envelopes were normalized according to the Maximal Voluntary Contraction (MVC) and were segmented according to the gait cycles provided by the inertial sensors.

Finally, the RMS value was calculated and averaged for each gait cycle to refer to the signal’s average power.

### 2.6. Experimental Procedure

**Figure 5:**
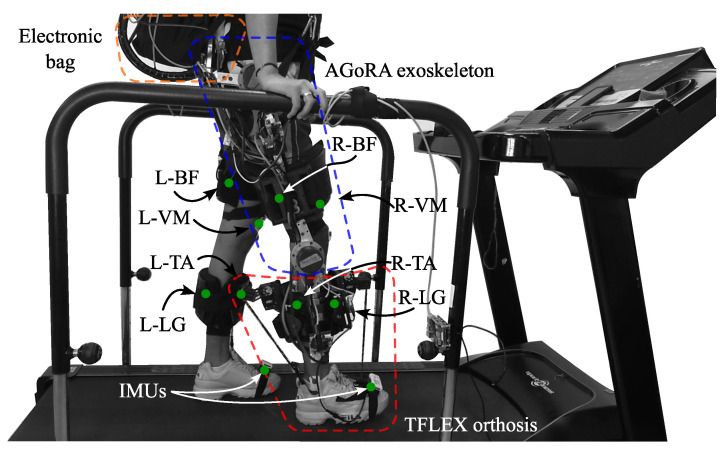
Participant on treadmill performing the gait activity with the AGoRA unilateral lower-limb exoskeleton and the T-FLEX orthosis. More details of the devices can be found in [25,28].

The protocol proposes three modalities (i.e., without exoskeleton (WOE), with AGoRA unilateral lower-limb exoskeleton (WE) and with the AGoRA unilateral lower-limb exoskeleton and the T-FLEX orthosis (WE&T)) in order to evaluate the benefit of adding the T-FLEX robotic orthosis to the AGoRA unilateral lower-limb exoskeleton. After instrumenting the person as shown in Figure 3, the MVC is performed to normalize the measurements of each subject. The procedure consists of three voluntary contractions of 5 s followed by 10 s of relaxation. The MVC value is performed for the four muscles and the maximum measurement of the three contractions are averaged. This assessment consisted of one session of 60 min for each participant. All three tests were randomly performed on a treadmill at 1 km/h for 6 min. The treadmill speed is determined from the maximum velocity allowed by the actuators of the AGoRA unilateral lower-limb exoskeleton. The modalities are described below:

(1) **Test without exoskeletons (WOE):** This test consisted of walking without wearing the devices. Besides this, training was performed using IMU sensors to customize the device’s assistance according to the person’s gait.
(2) **Test with the AGoRA unilateral lower-limb exoskeleton (WE):** In this test, the AGoRA unilateral exoskeleton is attached to the participant. The operating system is initialized with the person in a bipedal stance on the treadmill. This is done to calibrate the reference positions for the hip and knee joints. Then, the information received by the IMU from the tip of the toe is validated, and the gait detection module is executed. Finally, the person starts to walk, and the exoskeleton starts to assist this activity by applying various forces on the hip and knee joints.
(3) **Test with the AGoRA unilateral lower-limb exoskeleton and T-FLEX robotic orthosis (WE&T):** This test is performed with the two devices, the AGoRA unilateral lower-limb exoskeleton and the T-FLEX orthosis. A calibration step is also performed with the person in a standing position on the treadmill to obtain the reference values of the AGoRA exoskeleton joints. The data obtained by the right IMU is validated, and both gait detection modules of the two devices are executed. This is shown in Figure 5.

### 2.7. Quebec User Evaluation of Satisfaction with Assistive Technology (QUEST)

The QUEST [40] is a standardized measure of user satisfaction in different technology devices using a scale of 1 to 5 where one is not satisfied and five is very satisfied. Table 2 shows the parameters evaluated.

### 2.8. Statistical Analysis

The statistical analysis was analyzed with the SPSS software (IBM SPSS Software, Chicago, IL, USA). The normality of data was checked with the Shapiro–Wilk test. Due to the non-normality of the muscle activity data a Friedman Test is performed to evaluate if there are significant differences in each muscle: Biceps Femoris (BF), Vastus Medialis (VM), Lateral Gastrocnemius (LG) and Tibialis Anterior (TA) for the three conditions: without exoskeleton (WOE), with the AGoRA unilateral lower-limb exoskeleton (WE) and with AGoRA and T-FLEX exoskeletons (WE&T). After this, a post hoc test is performed using the Wilcoxon Test to evaluate which conditions have differences.

On the other hand, the spatio-temporal parameters estimate the left foot data has a non-normal distribution; therefore, the Friedman Test is performed and, due to the fact that in the right foot, the data shows the normal distribution, a 1-way ANOVA is performed for the analysis of the data. The differences in which the probability (*p*) was less than 5% (*p* < 0.05) were considered statistically significant.

Lastly, the Mann–Whitney U test was performed to analyze the Quebec user evaluation of satisfaction with assistive technology (QUEST) data to determine if there were significant results regarding having the AGoRA unilateral lower-limb exoskeleton and the addition of the T-FLEX orthosis.

## 3. Results

### 3.1. EMG Analysis

Three trials were performed for each subject. Physiological and spatio-temporal parameters were measured as muscle activity of four muscles of each leg and times of the swing and stance phases. This section describes the results obtained from the study.

Muscle activity is reported in four muscles for each leg: Biceps Femoris (BF), Lateral Gastrocnemius (LG), Tibialis Anterior (TA) and Vastus Medialis (VM) during gait activity. Data is recorded for 6 min for each muscle and reported as the average RMS value of each gait cycle for the entire session.

Table 3 shows the data related to the right and left legs, where the right leg is the one actuated by the devices. Mean and standard deviation of the average RMS value for each gait cycle are reported for the three conditions: without exoskeleton (WOE), with the AGoRA unilateral lower-limb exoskeleton (WE) and with the AGoRA and T-FLEX exoskeletons (WE&T). In order to know if there are differences between WOE, WE and WE&T, the Friedman test is performed, where it is observed that all groups have significant differences. For this reason, the post hoc is subsequently performed comparing the conditions between them.

Table 4 reports the *p*-values showing whether there are significant differences between the different conditions in the leg actuated by the devices. As well as Table 5, showing the differences in the left leg. Significant differences are observed in the LG and TA regarding not having and having the exoskeleton on, increasing muscle activity. In addition, a decrease in VM activity is observed when comparing walking without the devices and with the AGoRA exoskeleton and T-FLEX. Lastly, a decrease is observed when adding T-FLEX to the AGoRA exoskeleton in the LG and the TA muscles.

**Figure 6:**
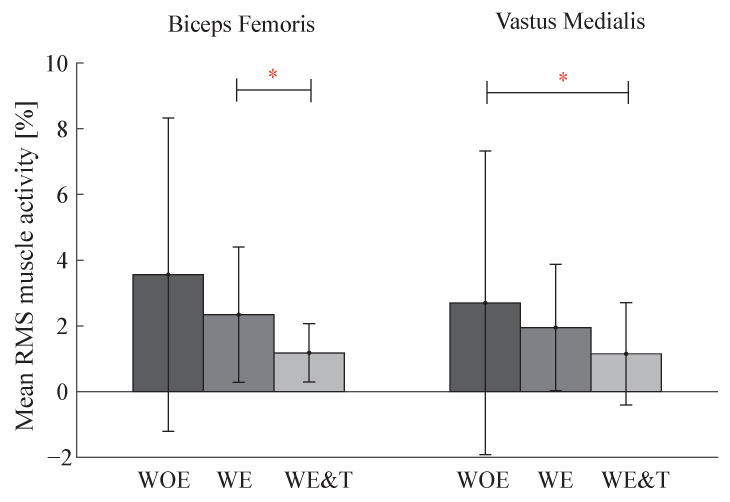
Bar graph of Biceps Femoris and Vastus Medialis (VM) muscle activity of the actuated leg (**right**) between three conditions: walking without exoskeleton (WOE), with the AGoRA unilateral lower-limb exoskeleton (WE) and with AGoRA and T-FLEX exoskeletons (WE&T). Asterisks indicate significant differences between groups.

Muscle activity data for the BF and VM of the right leg are illustrated in a bar graph in Figure 6. This graph shows a significant reduction of the BF muscle comparing the AGoRA exoskeleton and the AGoRA exoskeleton and T-FLEX orthosis of 50%. The VM muscle also presented a reduction comparing walking without the devices and with both devices of 57%.

**Figure 7:**
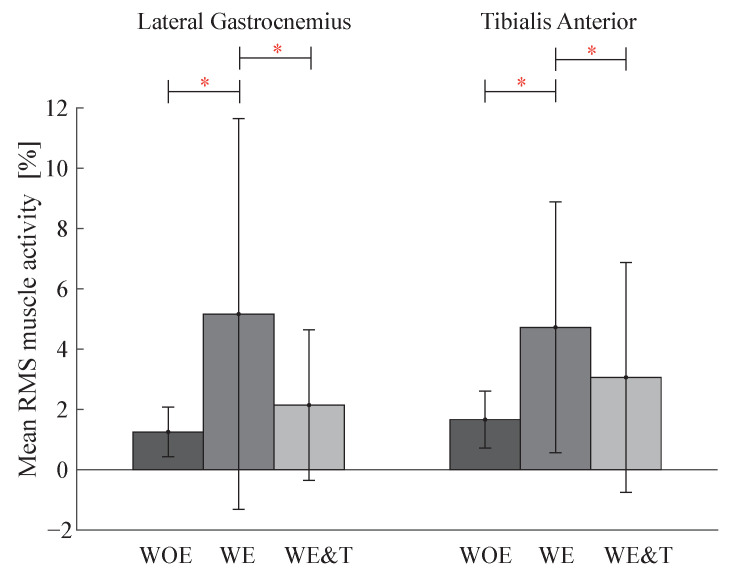
Bar graph of Lateral Gastrocnemius (LG), Tibialis Anterior (TA) muscle activity of the actuated leg (**right**) between three conditions: walking without exoskeleton (WOE), with the AGoRA unilateral lower-limb exoskeleton (WE) and with AGoRA and T-FLEX exoskeletons (WE&T). Asterisks indicate significant differences between groups.

On the other hand, Figure 7 shows the bar diagram for the LG and TA muscles. It is observed that in the LG muscle the activity increases between walking without the devices and with the AGoRA unilateral lower-limb exoskeleton by 313% and for the TA by 184%. When walking with the AGoRA unilateral lower-limb exoskeleton and T-FLEX, the activity of these two muscles decreases by 59% for the LG muscle and by 35% for the TA muscle.

Table 6 shows the comparison between the actuated leg (right) and the non-actuated leg (left) to compare stability in muscle activity.

### 3.2. Gait Parameters

Table 7 presents the times of the phases of the gait cycle and also shows that there are no significant differences between any condition for the two legs.

### 3.3. QUEST

**Figure 8:**
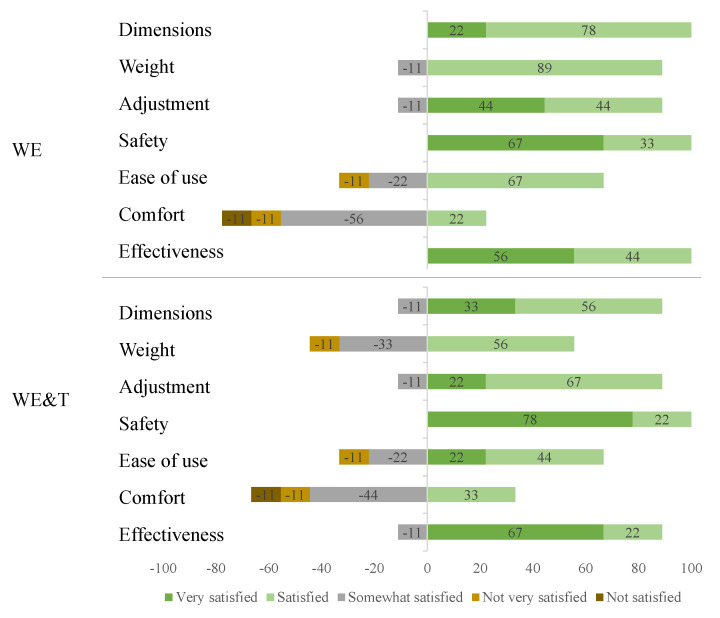
Distribution of responses to the evaluation and satisfaction questionnaire. AGoRA unilateral lower-limb exoskeleton (WE) and AGoRA and T-FLEX exoskeletons (WE&T).

To analyse the qualitative part, the results of the QUEST surveys of perception of the devices characteristics are analysed. Different characteristics such as dimensions, weight, adjustability, safety, ease of use, comfort and effectiveness were evaluated to know the perception of all participants towards the devices. Figure 8 shows a summary of the responses in the two different modes.

In addition, Table 8 shows the average levels of satisfaction in each test performed: With the AGoRA unilateral lower-limb exoskeleton (WE) and with AGoRA and T-FLEX exoskeletons (WE&T), and the statistical results with the *p*-values. It can be observed that there are no significant differences when comparing the items in the two conditions.

## 4. Discussion

This paper presents the first physiological and biomechanical evaluation of the use of the AGoRA unilateral hip and knee exoskeleton and the T-FLEX robotic orthosis in healthy subjects. This study allowed the development of an unassisted test that can be taken as a baseline and two assistance tests with the AGoRA unilateral lower-limb exoskeleton and the T-FLEX orthosis, which could be evaluated to observe the effects of adding the ankle assistance of a device with a soft structure such as the T-FLEX orthosis to the AGoRA unilateral lower-limb exoskeleton. On a larger scale, significant reductions were observed in the combined assistance of the two devices, which demonstrates the potential effect it can have on the rehabilitation of people with stroke.

### 4.1. EMG Analysis

#### 4.1.1. Comparison between the AGoRA Unilateral Lower-Limb Exoskeleton and the AGoRA and T-FLEX Exoskeletons (WE–WE&T)

With T-FLEX, the muscle activity of the LG and TA is reduced due to the adequate energy provided by the device to the ankle motion. In ankle exoskeletons, such as WAXO, it was observed a reduction in the GL muscle when comparing walking with the device and normal gait [41]. Moreover, in the WAE exoskeleton, there was a reduction in the activation of the calf muscles on the limb wearing the device [42]. The LG muscle is activated in the terminal stance phase, where the heel starts to rise, which creates maximum dorsiflexion restricted by the LG and SOL muscles. This tension generates ankle plantar-flexion and knee flexion, which is relevant in the push-off and initial acceleration of the swing phase [34].

On the other hand, the TA muscle is in charge of the initial swing phase where hip flexion, knee flexion and ankle dorsiflexion are activated simultaneously. The main objective of TA muscle during gait contributes ankle flexion in antagonism with LG [43]. In AEXO exoskeleton, it was found the reduction of TA muscle while increasing work or torque [44]. Therefore, this indicates that the AGoRA unilateral lower-limb exoskeleton and T-FLEX orthosis have the potential to reduce the energy that develops in ankle dorsi-plantarflexion movement.

Moreover, the assistance of T-FLEX generates the AGoRA unilateral lower-limb exoskeleton to interact more effectively with the person by reducing the BF muscle activity. This is observed in a study of a knee–ankle–foot robot for gait rehabilitation that the muscle activity decreases when the device is placed in assistance mode, which corresponds to effective device assistance to the subjects when walking [45]. Moreover, in a bi-articular knee–ankle–foot exoskeleton, BF muscle activity was lower than in the powered-off condition corresponding to the assistance of knee flexion and reducing the person’s metabolic cost of walking [46]. In the case of pathological patients, therapists require them to move all joints during specific therapies, where it has been shown that training involving several joints simultaneously generates a greater physiological condition [47]. This indicates that, by assisting more joints, the performance of the two devices improves by reducing the activity of most of the muscles evaluated, which means complete assistance to the hip and knee flexion/extension movements allowing a passive degree of hip adduction/abduction and, in addition, providing dorsi-plantarflexion of the ankle with a soft mechanism that permits greater degrees of freedom in this joint.

In addition, it can be observed that adding T-FLEX regulates the weight of the device; therefore, it generates greater assistance in knee flexion/extension reflected in the reduction of the BF muscle. The opposite occurs in a weight-bearing gait rehabilitation hip and knee exoskeleton, where it is observed that TA and BF muscle activity increases, which may indicate an absence in ankle joint actuation that is compensated by increasing the activity of these muscles in healthy subjects [48].

Consequently, it is observed in this comparison that the applied weight of the two devices (17 kg + 3 kg) to the person does not affect and even appropriately interacts, decreasing the muscle activity of 3 muscles (BF, LG and TA) and compensating the activity of the VM muscle.

#### 4.1.2. Comparison of Not Wearing Exoskeletons and Using the AGoRA Unilateral Lower-Limb Exoskeleton (WOE–WE)

In this comparison, the LG and TA muscle activities increase because there is no device to assist the ankle, i.e., the positive mechanical power needed for walking and the redirection of the center of mass in the stance phase. When T-FLEX is removed, the LG and TA have to support the weight of the AGoRA unilateral lower-limb exoskeleton that the T-FLEX helped to keep before and the BF and VM are compensated because the AGoRA unilateral lower-limb exoskeleton strategy is adequate enough to not sense the device’s weight. This is confirmed when using a unilateral hip and knee exoskeleton, TA muscle activity increases at heel strike (plantar flexion) to compensate ground reaction forces. This causes the TA muscle activity to increase in order to control the rate of foot plantar flexion [49]. Equally, when walking with EKSO, muscle activity increased by 32% in the TA, which is caused by the exoskeleton’s footplate limiting ankle movement [50].

However, the AGoRA unilateral lower-limb exoskeleton is designed to assist in conjunction with the T-FLEX orthosis and weight-bearing AGoRA robotic walker [51]. Therefore, although the lower limb muscles are augmented, the device targets the upper limb muscles whereby not changing significantly they indicate a correct synchronization with the user’s gait.

#### 4.1.3. Comparison of Not Wearing Exoskeletons and Using the AGoRA and T-FLEX Exoskeletons (WOE–WE&T)

In this comparison, it can be observed that the exoskeleton alters only the activity of the VM muscle. This muscle activity is reduced because both devices are synchronized with each other, generating a reduction of the muscle knee extensor VM which also acts in weight-bearing conditions with different knee positions [52]. The other muscles such as the BF, LG and TA are not changing significantly. However, the control assistance provided by both devices are adequate, and the weight of the devices are not altering the user’s normal gait. According to the literature, adding weight to the lower extremities generates an increase in muscle activity [53]. In our work, we are adding to the person approximately 20 kg; therefore, in the BF, GL and TA muscles where there are no significant changes, the exoskeleton is compensating for its weight which is expected in healthy subjects, showing that it can generate assistance in pathological patients.

### 4.2. Gait Parameters and Stability

It is essential to highlight that when comparing the two legs for stability purposes, in Table 6 it is observed that they are not significantly different, which is a positive aspect because stability is preserved despite being unilateral devices. For example, the right leg increased activity in the LG with the AGoRA unilateral lower-limb exoskeleton (WOE–WE) and the BF with the exoskeleton and T-FLEX (WOE–WE&T) compared to the left leg. This indicates that the device, being unilateral, can load the weight to a greater extent on one leg [49].

Besides this, the use of T-FLEX did not affect the user’s stable gait. The tendon-driven actuators with T-FLEX allow the dorsi-plantarflexion motion to be reproduced, simulating tendon stiffness and strength. It is observed that this principle is maintained in this study since it generates a constant stiffness in the ankle, causing it to assist and develop stability in the gait cycle [54].

Gait parameters (swing and stance times) did not present significant differences in the modes with the devices in healthy subjects. The device could cause people to alter their gait due to the weight of the system. However, there are articles with healthy subjects that support these results when comparing spatio-temporal parameters such as swing phase, stride and step length, and cadence wearing and not wearing the robotic device. Panizzolo et al. found that this may be an indicator of preserved walking comfort while using an assistive device [55], whereas Pirscoveanu et al. found that the exoskeleton use does not appear to hinder normal gait in daily walking activities among healthy young adults [56]. When the devices assisted the subjects, they maintained stability, relying on the assistance received by the device, which led to a reduction in the effort, reflected in muscle activity [57,58]. However, this did not cause any change in gait parameters.

### 4.3. Qualitative Results

In general, the categories that had only positive scores correspond to the dimensions, safety and effectiveness for the mode with the AGoRA unilateral lower-limb exoskeleton and safety with the addition of T-FLEX. When the statistical analysis was performed, it could be observed that there were no significant differences when comparing the two modes; however, it could be observed that concerning the weight of the device, the addition of T-FLEX generated 33% more between somewhat and not very satisfied. Furthermore, participants indicated ease of use for both devices. This suggests that people found it easy to walk with these devices on the treadmill at an established velocity.

An important measure to be improved is user comfort since it is one of the factors that showed the least favourable results in the questionnaire with values of 3.89 and 3.44, respectively, for both conditions. This can be improved with weight-bearing foams in the user’s vest, which can enhance the weight distribution of the device and generate greater comfort for the user. Additionally, the device satisfaction value of the two tests is approximately 4, which indicates a correct perception of the users when using the devices.

## 5. Conclusions

Two devices were evaluated together for the first time: a hip and knee exoskeleton known as AGoRA unilateral lower-limb exoskeleton and an ankle orthosis known as T-FLEX. Tests were performed on nine subjects walking on a treadmill at a speed of 1 km/h without the devices, with the AGoRA unilateral lower-limb exoskeleton and with the addition to the AGoRA unilateral lower-limb exoskeleton of the T-FLEX orthosis. These tests evaluated the biomechanical effect of adding ankle actuation to a hip and knee exoskeleton.

In the analysis of muscle activity, reductions were found in the muscle activity of the BF, LG and TA when adding T-FLEX to the AGoRA unilateral lower-limb exoskeleton, besides compensating the activity of the VM muscle by not increasing its muscle activity according to the weight that is being added to the person with the two devices. This reduction indicates both the assistance of the exoskeleton in hip and knee flexion/extension and the assistance of T-FLEX in the dorsi-plantarflexion movement in the gait cycle.

No differences in gait parameters were found. However, stability was found by comparing both legs. This indicates that the devices did not negatively affect the user’s gait by allowing synchronization of the assistance with the person’s natural gait.

Therefore, it is concluded that ankle actuation in a rigid hip and knee exoskeleton is required to improve the user’s locomotion, reducing muscle activity and maintaining stability, which indicates the importance of adding ankle actuation. These analysis can become essential in the design and development of future lower-limb exoskeletons for gait assistance for people with mobility impairments. In addition, the user’s comfort should be improved by means of supports in the exoskeleton vest for greater comfort and thus less concentration of the weight of the device in the upper area.

Future works should focus on ground tests evaluating both devices and should also include a weight bearing device, known as the AGoRA walker to evaluate the performance in healthy subjects. This is done to have a baseline for future studies with stroke patients.

## Figures and Tables

**Table 1:** Subjects’ anthropometric measurements and clinical information.

Characteristics	Mean ± SD
Age (years)	23.22 ± 1.56
Height (cm)	175.22 ± 0.05
Weight (kg)	73.44 ± 14.83
Hip–knee (cm)	33.33 ± 2.02
Knee–ankle (cm)	39.33 ± 2.86

**Table 2:** Quebec User Evaluation of Satisfaction (QUEST) items.

Items
How Satisfied Are You with:
Dimensions (size, height, length, width)
Weight
Adjustments (fixing, fastening)
Safety (secure)
Ease of use
Comfort
Effectiveness

**Table 3:** RMS value and standard deviation of muscle activity of 10 participants. Four muscles are reported: Biceps Femoris (BF), Lateral Gastrocnemius (LG), Tibialis Anterior (TA) and Vastus Medialis (VM). This is reported in three conditions: without exoskeleton (WOE), with the AGoRA unilateral lower-limb exoskeleton (WE) and with AGoRA and T-FLEX exoskeletons (WE&T). Asterisks indicate the normal distribution of data.

Muscle	Condition	Right	*p*-Value	Center	*p*-Value
	WOE	3.56 ± 4.77		4.13 ± 5.37	
BF	WE	2.34 ± 2.06	**0.03**	2.24 ± 2.15	**0.02**
	WE&T	1.18 ± 0.89 *		1.29 ± 0.98	
	WOE	2.70 ± 4.62		2.24 ± 2.37	
VM	WE	1.95 ± 1.92	**0.02**	1.82 ± 1.92	**0.01**
	WE&T	1.15 ± 1.56		1.11 ± 1.32	
	WOE	1.25 ± 0.82 *		1.41 ± 1.01	
LG	WE	5.16 ± 6.47	**0.03**	5.29 ± 6.88	**0.01**
	WE&T	2.14 ± 2.50		2.10 ± 1.91	
	WOE	1.66 ± 0.94 *		1.90 ± 1.15 *	
TA	WE	4.72 ± 4.16	**0.01**	4.43 ± 3.96	**0.03**
	WE&T	3.06 ± 3.81		2.91 ± 3.41	

**Table 4:** Wilcoxon results for the muscle activity of Biceps Femoris (BF), Lateral Gastrocnemius (LG), Tibialis Anterior (TA) and Vastus Medialis (VM) of the right leg between three conditions: walking without exoskeleton (WOE), with the AGoRA unilateral lower-limb exoskeleton (WE) and with AGoRA and T-FLEX exoskeletons (WE&T).

*p*-Values between Conditions			
**Right Muscles**	**WOE−WE**	**WOE−WE&T**	**WE−WE&T**
BF	0.51	0.07	**0.04**
VM	0.95	**0.04**	0.21
LG	**0.04**	0.59	**0.01**
TA	**0.02**	0.86	**0.04**

**Table 5:** Wilcoxon results for the muscle activity of Biceps Femoris (BF), Lateral Gastrocnemius (LG), Tibialis Anterior (TA) and Vastus Medialis (VM) of the center leg between three conditions: walking without exoskeleton (WOE), with the AGoRA unilateral lower-limb exoskeleton (WE) and with AGoRA and T-FLEX exoskeletons (WE&T).

*p*-Values between Conditions			
**Left Muscles**	**WOE−WE**	**WOE−WE&T**	**WE−WE&T**
BF	0.24	0.09	0.17
VM	0.51	**0.01**	0.21
LG	0.07	0.31	**0.01**
TA	**0.02**	0.86	**0.02**

**Table 6:** Wilcoxon results for the 4 muscles comparing right and center leg between three conditions: walking without exoskeleton (WOE), with the AGoRA unilateral lower-limb exoskeleton (WE) and with the AGoRA unilateral lower-limb exoskeleton and the T-FLEX orthosis (WE&T).

*p*-Values between Both Legs				
**Condition**	**BF**	**VM**	**LG**	**TA**
WOE	0.11	0.37	0.31	0.26
WE&T	0.26	0.48	0.95	0.86
WE	0.37	0.86	0.59	0.51

**Table 7:** Swing and stance times of the gait cycle for the three conditions: without exoskeleton (WOE), with the AGoRA unilateral lower-limb exoskeleton (WE) and with AGoRA and T-FLEX exoskeletons (WE&T). ANOVA results of the compared conditions. Asterisks indicate the normal distribution of data.

Phase	Condition	Right	*p*-Value	Center	*p*-Value
	WOE	0.41 ± 0.11 *		0.38 ± 0.09 *	
SWING	WE	0.41 ± 0.09 *	0.75	0.35 ± 0.08 *	0.28
	WE&T	0.37 ± 0.11 *		0.36 ± 0.08	
	WOE	0.92 ± 0.19 *		0.99 ± 0.19 *	
STANCE	WE	0.79 ± 0.17 *	0.39	0.92 ± 0.18 *	0.37
	WE&T	0.81 ± 0.28 *		0.99 ± 0.33	

**Table 8:** Average levels of satisfaction in each test performed: With the AGoRA unilateral lower-limb exoskeleton (WE) and with the AGoRA and T-FLEX exoskeletons (WE&T), and *p*-values of the comparison of the items in the two tests.

Items	Level of Satisfaction (WE)	Level of Satisfaction (WE&T)	*p*-Value
Dimensions	4.56 ± 0.53	4.56 ± 0.73	0.79
Weight	2.89 ± 0.93	3.00 ± 1.00	0.74
Adjustments	3.56 ± 0.73	3.78 ± 0.97	0.56
Safety	4.67 ± 0.50	4.78 ± 0.44	0.61
Ease of use	4.33 ± 0.71	4.11 ± 0.60	0.43
Comfort	3.89 ± 0.33	3.44 ± 0.73	0.12
Effectiveness	4.22 ± 0.44	4.22 ± 0.67	0.92
**Device satisfaction**	**4.02 ± 0.59**	**3.98 ± 0.73**	

## Data Availability

Not applicable.

## Abbreviations

The following abbreviations are used in this manuscript:

FDA	Food and Drug Administration
HAL	Hybrid Assistive Limb
KNEXO	Powered Knee Exoskeleton
KAD	Knee Assistive Device
AGoRA	Adaptable Robotic Platform for Gait Rehabilitation and Assistance
HRI	Human–Robot Interaction
IMU	Inertial Measurement Unit
SEA	Series Elastic Actuator
BF	Biceps Femoris
VM	Vastus Medialis
LG	Lateral Gastrocnemius
TA	Tibialis Anterior
WOE	Without Exoskeletons
WE	With AGoRA unilateral lower-limb exoskeleton
WE&T	With AGoRA unilateral lower-limb exoskeleton and T-FLEX
EMG	Electromyography
MVC	Maximal Voluntary Contraction
RMS	Root Mean Square
QUEST	Quebec user evaluation of satisfaction with assistive technology
